# Relationship Between Glycosylated Hemoglobin Variability and the Severity of Coronary Artery Disease in Patients With Type 2 Diabetes Mellitus

**DOI:** 10.1155/2024/9958586

**Published:** 2024-08-01

**Authors:** Xinyan Liu, Xiyao Yang, Na Wu

**Affiliations:** ^1^ Department of Endocrinology Shengjing Hospital of China Medical University, Shenyang 110004, China; ^2^ Department of Endocrinology The First Hospital of China Medical University, Shenyang 110004, China; ^3^ Department of Pediatrics Shengjing Hospital of China Medical University, Shenyang 110004, China

**Keywords:** coronary heart disease, diabetic cardiovascular complications, glycosylated hemoglobin variability, HbA1c time in range, Type 2 diabetes mellitus

## Abstract

**Background:** Glycosylated hemoglobin (HbA1c) variability is a risk factor for cardiovascular complications in patients with Type 2 diabetes mellitus (T2DM), but its relationship with the severity of coronary artery disease (CAD) is unclear.

**Methods:** Patients with T2DM who underwent coronary angiography due to angina were enrolled. HbA1c variability was expressed as coefficient of variation (CV), standard deviation (SD), variability independent of mean (VIM), and time in range (TIR). The severity of CAD was expressed by the number of involved vessels and Gensini score. Multivariate regression models were constructed to test the relationship between HbA1c variability, number of involved vessels, and the Gensini score, followed by linear regression analysis.

**Results**: A total of 147 patients were included. In multivariate analysis, VIM-HbA1c (OR = 2.604; IQR: 1.15, 5.90; *r* = 0.026) and HbA1cTIR (OR = 0.13; IQR: 0.04, 0.41; *r* < 0.001) were independent risk factors for the number of involved vessels. After adjustment, HbA1cTIR (OR = 0.01; IQR: 0.002, 0.04; *r* < 0.001), SD-HbA1c (OR = 4.12, IQR: 1.64, 10.35; *r* = 0.001), CV-HbA1c (OR = 1.41, IQR: 1.04, 1.92; *r* = 0.007), and VIM-HbA1c (OR = 3.26; IQR: 1.43, 7.47; *r* = 0.003) were independent risk factors for the Gensini score. In the linear analysis, the Gensini score was negatively correlated with HbA1cTIR (*β* = −0.629; *r* < 0.001) and positively correlated with SD-HbA1c (*β* = 0.271; *r* = 0.001) and CV-HbA1c (*β* = 0.176; *r* = 0.033). After subgroup analysis, HbA1cTIR was a risk factor for the number of involved vessels. The Gensini score was negatively correlated with HbA1cTIR and positively correlated with SD-HbA1c at subgroups of subjects with a mean HbA1c ≤ 7%.

**Conclusions:** Our analysis indicates that HbA1c variability, especially HbA1cTIR, plays a role for the severity of CAD in patients with T2DM. HbA1c variability may provide additional information and require management even at the glycemic target.

**Translational Aspects:** Studies have shown that HbA1c variability is related to cardiovascular complications. Further, we explore the correlation between HbA1c variability and the severity of CAD. HbA1c variability is a risk factor for coronary stenosis in T2DM. It may be a potential indicator reflecting glycemic control for the prevention and treatment of cardiovascular complications.

## 1. Introduction

With the improvement of living standard, the prevalence of diabetes is increasing year by year. According to the latest epidemiological survey and research, the overall prevalence of diabetes in mainland China is 11.2%, of which Type 2 diabetes mellitus (T2DM) accounts for more than 90% [1]. Diabetes has become a global public health problem and danger to human health [2]. Compared with healthy individuals, patients with T2DM have a higher risk of cardiovascular disease [3], and cardiovascular complications are the leading cause of morbidity and mortality in these patients.

Glycosylated hemoglobin (HbA1c), as an indicator that reflects the average blood glucose level over the past 3 months, is recommended as a gold standard in blood glucose management. The American Diabetes Association (ADA) suggested that HbA1c ≤ 7.0% may help delay the occurrence and development of diabetic complications [4]. However, recent clinical trials of intensive glycemic control in patients with Type 2 diabetes did not demonstrate beneficial effects on cardiovascular complications [5–7]. This suggests that focusing only on HbA1c standard may have limitations [8].

Previous studies have suggested that three main glycemic abnormalities, including hyperglycemia, hypoglycemia, and glucose variability (GV), may all accelerate the progression of cardiovascular complications in patients with T2DM [9]. In recent years, growing attention has been paid to the possible role of GV in the development of diabetic complications, particularly cardiovascular complications. Glucose variability includes short-term and long-term glucose variability. Previously, studies suggested that short-term glucose variability may be a risk factor for cardiovascular complications [10–12]. Other studies have suggested that long-term glucose variability, reflected by the variability of HbA1c and fasting blood glucose (FBG) over a period of time, can reflect the glucose variability over a longer period of time and is related to cardiovascular complications and all-cause mortality [13–18].

At present, glucose variability is mostly expressed by standard deviation (SD) and coefficient of variation (CV). The international consensus proposed that the time in range (TIR), defined as the percentage of time within 70–180 mg/dL (3.9–10 mmol/L), could reflect short-term glucose variability and blood glucose control and may minimize the risk of complications caused by hypoglycemia and hyperglycemia [19]. Prentice et al. suggested that HbA1cTIR, defined as the percentage of time within the target range of HbA1c, could reflect HbA1c variability and may be an independent risk factor for cardiovascular complications and all-cause mortality in patients with T2DM [20].

The severity of coronary artery disease (CAD) may affect the prognosis; however, studies on the relationship between long-term glucose variability and the severity of CAD in patients with T2DM are limited. Some studies suggested that the HbA1c may be associated with the severity of CAD in patients with T2DM [21–23]. However, some studies have also shown that HbA1c level is not related [24]. Currently, there are a variety of scoring systems used for quantitative analysis of coronary artery lesions, and the Gensini score is more commonly used in clinical practice. The Gensini score fully considers the number, location, and degree of stenosis of coronary artery lesions, which is a more scientific evaluation standard of coronary artery lesions. At the same time, this scoring system has also been widely used in related studies on the clinical outcomes of CAD [25]. In this study, we further explore the relationship between HbA1c variability and the severity of CAD (assessed by the Gensini score and the number of involved vessels) in order to improve the survival of patients with T2DM.

## 2. Methods

### 2.1. Study Participants

**Figure 1:**
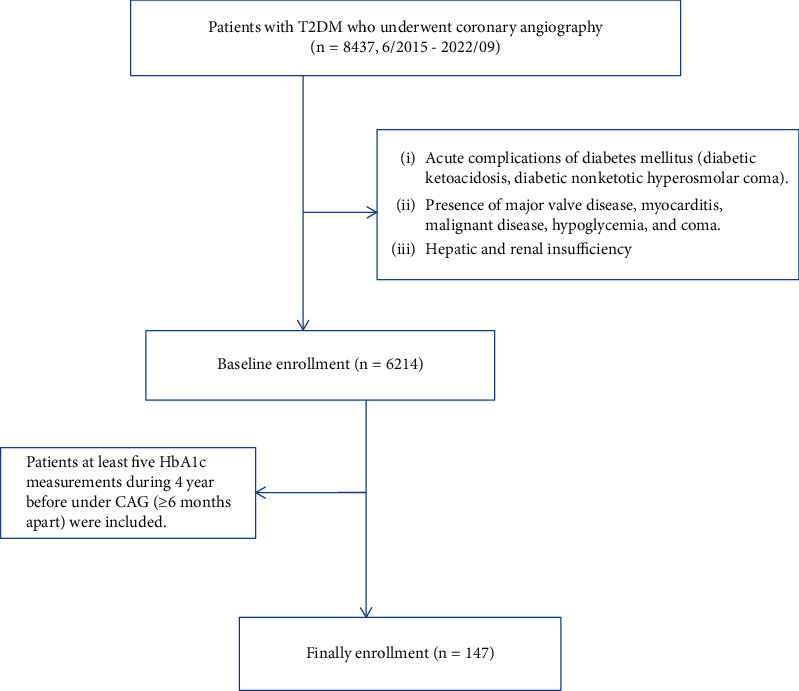
Flow chart of patient enrollment.

We conducted a retrospective study of participants from patients with T2DM who underwent coronary arteriography (CAG) due to angina and were enrolled at Shengjing Hospital Affiliated with China Medical University from June 2015 to September 2022. The inclusion criteria for patients were as follows (*n* = 8437): (1) men and women over 18 years old, (2) patients diagnosed with T2DM according to the 2020 edition of the guideline for the prevention and treatment of Type 2 diabetes in China, and (3) patients who underwent coronary angiography. Exclusion criteria were as follows (*n* = 2223): (1) acute complications of diabetes mellitus (diabetic ketoacidosis, diabetic nonketotic hyperosmolar coma); (2) presence of major valve disease, myocarditis, malignant disease, hypoglycemia, and coma; and (3) hepatic and renal insufficiency. For calculation of HbA1c variability, subjects without at least five HbA1c measurements during the 4 years before under CAG (≥ 6 months apart) were further excluded (*n* = 6067). Thus, 147 patients comprised the final enrollment (as shown in Figure 1). This study was approved by the ethics committee of Shengjing Hospital (ethics number 2022PS699K).

### 2.2. Clinical and Biochemical Data Assessments

The basic information and clinical characteristics of the participants were collected, including age, height, systolic blood pressure (SBP), diastolic blood pressure (DBP), the duration of diabetes, smoking habits, and use of insulin. The body mass index (BMI) was calculated based on height and weight. Besides HbA1c levels, we also collected other laboratory information, including levels of blood lipids such as triglycerides and low-density lipoprotein cholesterol (LDL-C) and estimated glomerular filtration rate (EGFR). After patients underwent selective CAG, the angiographic results were collected. Each coronary artery was displayed on at least two different planes.

### 2.3. Evaluation of HbA1c Variability

HbA1c variability was expressed as SD-HbA1c, CV-HbA1c, VIM-HbA1c, and HbA1cTIR during the 4 years before under CAG. We determined the SD-HbA1c of several HbA1c measurements for each patient. We calculated the CV-HbA1c, defined as CV=SD-HbA1c/meanHbA1c. In addition, we evaluated the variation independent of mean value (VIM-HbA1c), defined as 100 × SD/mean *β*, where *β* is the regression coefficient, the natural log (ln) of the SD over the ln of the mean. In addition, we evaluated HbA1c variability by HbA1cTIR and the percentage of time that HbA1c levels were within the target range. To calculate HbA1cTIR [20], we first set the HbA1c target range for each patient individually according to expert consensus on HbA1c targets and management algorithms for Chinese adults with T2DM [26] (as shown in Table S1). These values are updated every year, considering the age of the patients and the development of new conditions and diabetic complications. Finally, we computed HbA1cTIR as a percentage of time that HbA1c levels were within the target range [20]. In addition, SD-HbA1c, CV-HbA1c, and HBA1cTIR may reflect the variability and the mean level of long-term blood glucose levels. The application of TIR can help reduce the risk of hypoglycemia and hyperglycemia. And HbA1cVIM may reflect the variability of HBA1c and is not related to the mean level of HBA1c. Therefore, we also discuss the relationship between these two indicators and the severity of CAD in this article.

### 2.4. Assessment of Severity of CAD

We used two methods to evaluate the severity of CAD, the Gensini score and the number of involved vessels [25]. The number of involved vessels was characterized as 1-vessel disease, 2-vessel disease, and 3-vessel disease. We evaluated the Gensini score, which assigned the severity score based on the degree and location of stenosis based on previous publications. In detail, the narrowing was scored as 32 for 100% occluded artery, 16 for 91%–99%, 8 for 76%–90%, 4 for 51%–75%, 2 for 26%–50%, and 1 for 1%–25%. The score was then multiplied by a coefficient to indicate the functional importance of each segment. The coefficient was as follows: the left main coronary artery was five; the proximal left anterior descending artery and the proximal circumflex artery were 2.5; the middle left anterior descending artery was 1.5; the right coronary artery, the distal left anterior descending artery, the posterolateral artery, and the obtuse artery were 1.0; and the residual major segments were 0.5 [25]. Finally, the Gensini score was calculated by the sum of the integral of each coronary artery segment.

### 2.5. Statistical Methods

Descriptive data are summarized as median and interquartile range for continuous variables and percentages for categorical variables. All patients were divided into three groups according to the number of involved vessels: 1-vessel disease, 2-vessel disease, and 3-vessel disease. For normally distributed variables, differences in different groups were analyzed by analysis of variance (ANOVA). For nonnormally distributed continuous variables, differences were analyzed by the Mann–Whitney *U* test or the Kruskal–Wallis test. Then, we divided all patients into three groups according to the Gensini score, and we analyzed the differences between the characteristics of participants in the different groups.

Further, we divided the patients according to tertiles of HbA1c variability. We analyzed the difference in the number of involved vessels and Gensini scores between different groups.

The association between HbA1c variability and risk of the severity of CAD was investigated through multivariate logistic regression analyses. Logistic regression analysis was used to estimate odds ratios (ORs) of HbA1c variability and the number of involved vessels. In Model 1, no factors were adjusted. Model 2 was adjusted for sex, age, duration of diabetes, SBP, DBP, LDL, and the use of insulin. Model 3 was adjusted for sex, age, duration of diabetes, SBP, DBP, LDL, use of insulin, and HbA1c level. Logistic regression analysis was used to estimate ORs of HbA1c variability and the Gensini score. In Model 1, no factors were adjusted. In Model 2, sex, age, duration of diabetes, SBP, DBP, LDL, and the use of insulin were adjusted. In Model 3, sex, age, duration of diabetes, SBP, DBP LDL, use of insulin, and HbA1c were adjusted. Linear regression analysis was performed to determine the relationship between HbA1c variability and the Gensini score. To avoid collinearity between these parameters, each parameter had been in separated model.

Subjects were also divided into two subgroups, based on the average HbA1c levels ≤ 7% or > 7%. The relationship between HbA1c variability and the number of involved vessels was analyzed by multivariate regression. The relationship between HbA1c variability and the Gensini score was analyzed by linear regression and multivariate regression in the different subgroups.

All analyses were performed using SPSS version 25.0 for Windows (SPSS Corporation, Chicago, Illinois, USA). A *r*-value of < 0.05 was considered statistically significant. Continuous variables are presented as median (interquartile range) or mean ± SD and categorical data are summarized as frequencies (percentages). For normally distributed variables, differences in different groups were analyzed by ANOVA. For nonnormally distributed continuous variables, differences were analyzed by the Mann–Whitney *U* test or the Kruskal–Wallis test.

## 3. Results

### 3.1. Basic Characteristics of the Study Population

From the 8437 T2DM patients under coronary angiography in the database, we identified all subjects with at least five measurements of HbA1c taken over a period of 4 years before under CAG (*n* = 147). The mean number of HbA1c tests in the entire population was 5.41.

Compared with patients with 1-vessel disease, the duration of diabetes, use of insulin, SD-HbA1c, and HbA1c were increased, and HbA1cTIR was decreased in patients with multiple vessel disease. There were no significant differences in age, sex, smoking habits, SBP, DBP, LDL, triglyceride, EGFR, BMI, the number of HbA1c measurements CV-HbA1c, and VIM-HbA1c (as shown in Table 1(a)).

Compared with the lowest quartile (Q1) groups of the Gensini score, disease duration, insulin use, SD-HbA1c, and CV-HbA1c were increased, and HbA1cTIR was decreased in higher groups (as shown in Table 1(b)). There were no significant differences in age, sex, smoking habits, SBP, DBP, BMI, the number of HbA1c, triglyceride, EGFR, VIM-HbA1c, or LDL between the groups.

### 3.2. Associations of HbA1c Variability With Severity of CAD

Coronary artery stenosis was compared in subjects stratified by tertiles of HbA1C variability. The percentages of 3-vessel, multivessel, and Gensini scores decreased with increasing tertiles of HbA1cTIR. Gensini scores increased with increasing tertiles of SD-HbA1c and CV-HbA1c (as shown in Table S2).

Univariate and multivariate regression analyses were performed to analyze the association between the coronary artery stenosis and different measures of HbA1c variability. In univariate logistics analysis, HbA1cTIR (OR, 0.09; IQR: 0.03, 0.24; *r* < 0.001) and SD-HbA1c (OR, 2.59; IQR: 1.32, 5.11; *r* = 0.007) were risk factors for the number of involved vessels. After adjusting risk factors, such as sex, age, duration, HbA1c, SBP, DBP, LDL, and use of insulin, HbA1cTIR (OR, 0.13; IQR: 0.04, 0.41; *r* < 0.001) and VIM-HbA1c (OR, 2.60; IQR: 1.15, 5.90; *r* = 0.026) were independent risk factors for the number of involved vessels, and the difference was statistically significant (as shown in Table 2).

In univariate logistics analysis, HbA1cTIR (OR, 0.007; IQR: 0.002, 0.03; *r* < 0.001), SD-HbA1c (OR, 4.15; IQR: 2.03, 8.48; *r* < 0.001), CV-HbA1c (OR, 1.42; IQR: 1.08, 1.86; *r* = 0.004), and VIM-HbA1c (OR, 2.10; IQR: 1.01, 4.36; *r* = 0.036) were risk factors for the Gensini score. After adjusting risk factors, such as sex, age, duration, HbA1c, SBP, DBP, LDL, and use of insulin using HbA1cTIR (OR, 0.01; IQR: 0.002, 0.04; *r* < 0.001), SD-HbA1c (OR, 4.12; IQR: 1.64, 10.35; *r* = 0.001), CV-HbA1c (OR, 1.41; IQR: 1.04, 1.92; *r* = 0.007), and VIM-HbA1c (OR, 3.26; IQR: 1.43, 7.47; *r* = 0.003) were independent risk factors for the Gensini score, and the difference was statistically significant (as shown in Table S3).

**Figure 2:**
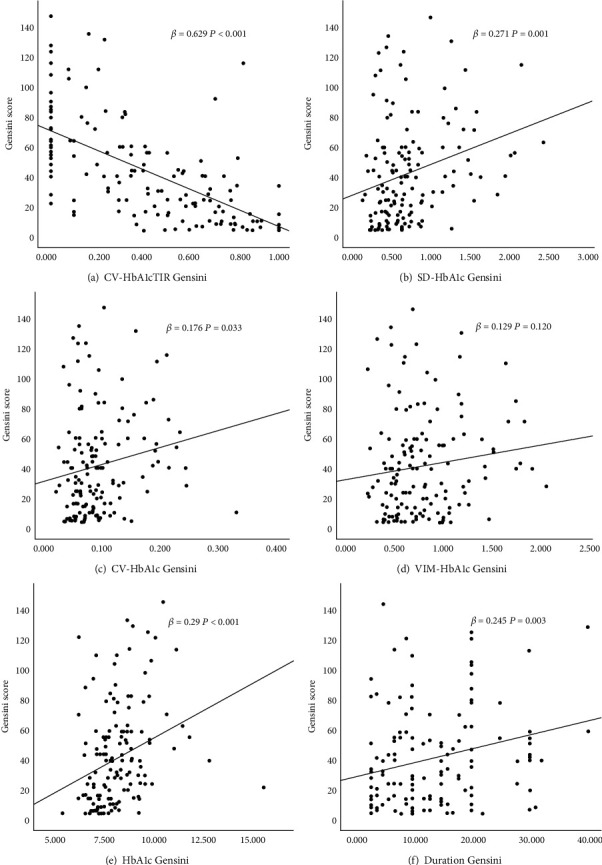
Associations between the Gensini score and HbA1c variability by univariate linear regression analysis. The Gensini score was negatively correlated with HbA1cTIR ((a) *β* = −0.629; *r* = 0.001) and positively correlated with SD-HbA1c ((b) *β* = 0.271; *r* = 0.001), CV-HbA1c ((c) *β* = 0.176; *r* = 0.031), HbA1c ((e) *β* = 0.29; *r* < 0.001), and duration ((f) *β* = 0.248; *r* = 0.02). (d) The Gensini score was not correlated with HbA1cVIM.

Univariate and multivariate linear regression analyses were performed to analyze the association between the Gensini score and HbA1c variability. Univariable linear regression analysis showed that Gensini score was negatively correlated with HbA1cTIR (*β* = −0.629; *r* < 0.001) and positively correlated with SD-HbA1c (*β* = 0.271; *r* = 0.001), CV-HbA1c (*β* = 0.176; *r* = 0.033), HbA1c (*β* = 0.29; *r* < 0.001), and duration (*β* = 0.245; *r* = 0.002). (as shown in Table 3 and Figure 2). After adjusting for factors such as age, sex, duration, SBP, DBP, LDL, insulin use, and HbA1c, the Gensini score was negatively correlated with HbA1cTIR (*β* = −0.576; *r* < 0.001) and positively correlated with SD-HbA1c (*β* = 0.217; *r* = 0.024) and CV-HbA1c (*β* = 0.160; *r* = 0.031) (as shown in Table 3).

### 3.3. Subgroup Analysis of the Relationship Between HbA1c Variability and Severity of CAD

To assess if GV was associated with the degree of coronary artery stenosis also in subjects at target (AT) HbA1c, we analyzed the subgroups of patients with mean HbA1c ≤ 7% (AT) or > 7% (not at target [NAT]). After subgroup analysis, in the HbA1c ≤ 7% group, HbA1cTIR (OR, 0.008; IQR: 0.001, 0.08; *r* < 0.001) was an independent risk factor for the number of involved vessels. The Gensini score was negatively correlated with HbA1cTIR (*β* = −0.713, *r* < 0.001) (as shown in Tables S4a and S4b). At HbA1c > 7%, the Gensini score was negatively correlated with HbA1cTIR (*β* = −0.576; *r* < 0.001) and positively correlated with SD-HbA1c (*β* = 0.215; *r* = 0.037) (as shown in Tables S4c and S4d).

## 4. Discussion

The primary findings of our study are as follows. First, the Gensini score was negatively correlated with HbA1cTIR and positively correlated with CV-HbA1c and SD-HbA1c in patients with T2DM. Second, we found that HbA1cTIR and VIM-HbA1c are independent risk factors for the number of involved vessels. Third, HbA1cTIR is a risk factor for the severity of CAD in patients with T2DM, regardless of whether the mean blood glucose is within the target (HbA1c ≤ 7%). Overall, our results support that HbA1c variability, especially HbA1cTIR, may be related to the severity of CAD in patients with T2DM and is worthy of attention in blood glucose management.

In recent years, a number of studies have shown the association between long-term glucose variability and cardiovascular complications [13–18]. Meta-analysis reports also suggest that long-term glucose variability in patients with T2DM is associated with almost all cardiovascular complications. Although the mean level remains important for the prevention of cardiovascular events in patients with T2DM, previous studies suggested that HbA1c variability is an independent risk factor for cardiovascular disease regardless of being at the glycemic target [27]. In addition, Yang et al. [28] considered that HbA1c variability was an independent predictor of the incidence of in-stent restenosis in patients with Type 2 diabetes after stent implantation.

All the above studies suggested that long-term glucose variabilities were independent risk factors for cardiovascular disease in patients with T2DM, and targets in blood glucose management need to focus on both stability and absolute level of HbA1c [29]. However, the relationship between HbA1c variability and severity of CAD in patients with T2DM is not clear. Our experiment further explored the relationship between the HbA1c variability and the severity of CAD in patients with T2DM. Our results suggested that in patients with T2DM, HbA1cTIR and SD-HbA1c were risk factors for the number of involved vessels. After adjusting the risk factors, we found that the HbA1cTIR and VIM-HbA1c were independent risk factors for the number of involved vessels. After adjusting for related risk factors such as HbA1c, the logistics results still suggest that HbA1c variability was an independent risk factor for the Gensini score. After linear regression analysis, the results suggested that SD-HbA1c and CV-HbA1c were positively correlated with the Gensini score, while HbA1cTIR was negatively correlated with the Gensini score. Both the Gensini score and the number of involved vessels are indicators reflecting the severity of CAD. Our study has shown that the HbA1c variability may be a risk factor for the severity of CAD in patients with T2DM.

To analyze the reasons, it may be that patients with higher HbA1c variability may have poor management of blood glucose and poor basic vascular condition. In addition, hypoglycemia may mediate the association between HbA1c variability and severity of CAD. Recently, studies have also reported that long-term glucose variability is related to cardiovascular disease in patients within the target of HbA1c [30]. Other study suggests that glucose variability may predict the risk of hypoglycemia, especially in patients receiving intensive therapy [31]. After subgroup analysis, our study also suggests that HbA1cTIR was a risk factor for the severity of coronary artery stenosis in patients within the target of HbA1c [31, 32]. Our research suggested that HbA1c variability is related to the severity of CAD in patients with T2DM. Thus, in addition to the mean blood glucose, long-term stability is necessary for patients with diabetes in clinical practice. In addition, our study also suggested that setting the HbA1c target range for each patient individually may also be necessary for patients with diabetes in clinical practice. For patients with T2DM, we suggested the patients to maintain long-term stability of blood glucose and achieve individual target by lifestyle education and pharmacological tools.

The underlying mechanism of the relationship between HbA1c variability and cardiovascular disease in patients with T2DM is unknown. Oxidative stress, low-grade inflammation, and endothelial dysfunction may be the key drivers. Previous studies suggested that compared with chronic hyperglycemia, glucose variability is more likely to include vascular damage and endothelial dysfunction mainly mediated by oxidative stress [33–35]. Tateishi et al. suggest that GV is significantly associated with coronary rather than peripheral endothelial dysfunction in patients with CAD, which may be a part of underlying mechanisms of coronary atherosclerosis [36]. Glucose variability may lead to the accumulation of glycolytic intermediates, including the activation of the polyol pathway, advanced glycation end products, protein kinase C pathway, and hexosamine pathway, and may also participate in the damage of endothelial cells [37, 38]. Some studies have found that the oxidative stress of protein kinase C may cause the generation of reactive oxygen species (ROS) induced by activation. Vascular cell growth and apoptosis, extracellular matrix synthesis, and changes in vascular homeostasis further promote the development of atherosclerosis [38]. Long-term GV may also cause irreversible epigenetic modifications, resulting in *β*-cell dysfunction. Finally, hypoglycemia may lead to an increased risk of cardiovascular disease because of the release of inflammatory cytokines, increased platelet activation and endothelial dysfunction, and sympathetic-adrenal response (which may lead to arrhythmia and increased cardiac workload) [39–41]. Thus, adverse cardiovascular outcomes may result from a combination of these risk factors.

This study explored the relationship between HbA1c variability and the severity of CAD in patients with T2DM, which was rare in previous studies. For patients with diabetes, HbA1c is important in the blood glucose management. Our study suggested that in addition to mean HbA1c, HbA1c variability is also essential. The calculation and management for HbA1c variability are also convenient. At the same time, a relatively new indicator, HbA1cTIR, was introduced to minimize the risk of hypoglycemia, hyperglycemia, and complications during blood glycemic management. Thus, our study is rational and positive in clinical application.

This study has several limitations. First, despite our effort to ensure consistency in screening intervals, the number, frequency, and interval of HbA1c measurements vary from patient to patient. Second, our retrospective analysis selected patients who received treatment for coronary heart disease. Thus, many patients did not regularly accept diabetes management before this treatment and did not meet our requirements for HbA1c measurements, leading to a small sample size. Due to the same reason, our study lacks data on the prevalence of usage of the most relevant groups of medications and lifestyle-related surveys. In the further prospective studies, we will provide the prevalence of usage of the most relevant groups of medications. Further prospective studies are needed to investigate the possible causal relationship between the HbA1c variability and the severity of CAD in patients with T2DM and to calculate cut-off values for the HbA1c variability parameter. So that may help to explore whether the HbA1c variability is a valuable therapeutic target.

## 5. Conclusion

In conclusion, our findings suggest that HbA1c variability, especially HbA1cTIR, may be associated with the severity of coronary artery stenosis in patients with T2DM. This study suggests that in addition to mean HbA1c, long-term blood glucose variability is also important in blood glucose management, which deserves our attention and further exploration. In addition, these results support the necessity for using a personalized approach to HbA1c target and stability, especially in older patients with diabetes.

## Figures and Tables

**(a):** 

	**1-vessel disease (*n* = 42)**	**2-vessel disease (*n* = 56)**	**3-vessel disease (*n* = 49)**	**r**
Age (y)	62.07 ± 8.86	63.80 ± 9.95	66.49 ± 12.22	0.097
Sex, male (%)	23 (54.8%)	35 (62.5%)	30 (61.22%)	0.567
The number of HbA1c measurements	5.4 ± 0.14	5.4 ± 0.12	5.43 ± 0.13	0.90
Smoking habits (%)	12 (28.6%)	29 (51.79%)	20 (40.82%)	0.07
Duration (y)	5 (9. 12)	10 (7, 16)	18 (9, 20)^a^^,^^b^	0.001
SBP (mmHg)	134.85 ± 13.81	134.57 ± 18.09	138.18 ± 24.27	0.661
DBP (mmHg)	80.12 ± 10.19	76.82 ± 10.34	76.55 ± 11.82	0.157
LDL (mmol/L)	2.01 (1.61, 2.66)	2.06 (1.48, 2.77)	2.04 (1.5, 2.49)	0.894
EGFR (mL/min/1.73 m^2^)	95.83 ± 4.49	89.58 ± 3.89	79.98 ± 4.08	0.29
Triglyceride (mmol/L)	2.19 ± 0.32	2.32 ± 0.31	1.59 ± 0.12	0.7
Insulin (%)	18 (41.86%)	30 (53.57%)	37 (75.5%)^a^^,^^b^	0.006
BMI (kg/m^2^)	25.70 (23.83, 27.3)	24.52 (23.00, 26.78)	24.50 (22.90, 25.95)	0.213
HbA1c	6.98 (6.52, 7.66)	7. 18 (6.66, 8.04)	7.88 (7.28, 8.70)^a^^,^^b^	0.001
HbA1cTIR	0.7 (0.41, 0.90)	0.45 (0.10, 0.67)^a^	0.21 (0, 0.47)^a^^,^^b^	< 0.001
SD-HbA1c	0.43 (0.32, 0.71)	0.55 (0.34, 0.84)	0.65 (0.43, 1)^a^	0.017
CV-HbA1c	0.07 (0.05, 0.10)	0.07 (0.05, 1.00)	0.09 (0.06, 0.13)	0.127
VIM-HbA1c	0.59 (0.43, 0.953)	0.66 (0.47, 0.82)	0.72 (0.53, 1.04)	0.254

*Note:* The continuous variables were expressed as mean standard deviation, and the comparisons were made by analysis of variance. If the data did not conform to the normal distribution, it was expressed as the median (interquartile range) and compared with the nonparametric test. Categorical variables were expressed as frequency (percentage) and compared using a chi-square test. Values are expressed as *n* (%) or median (interquartile range).

Abbreviations: BMI, body mass index; CV, coefficient of variation; DBP, diastolic blood pressure; EGFR, estimated glomerular filtration; Hb1Ac, glycosylated hemoglobin; HbA1cTIR, HbA1c time in range; LDL, low-density lipoprotein cholesterol; SBP, systolic blood pressure; SD, standard deviation; VIM, variation independent of mean.

^a^Statistically significant difference compared to the first group.

^b^Statistically significant difference compared to the second group.

**(b):** 

	**T1 (*n* = 50)**	**T2 (*n* = 47)**	**T3 (*n* = 50)**	**p**
Age (y)	63.94 ± 7.70	61.76 ± 11.43	66.57 ± 11.90	0.058
Sex, male (%)	27 (50%)	29 (61.7%)	31 (62%)	0.658
Smoking habits (%)	19 (38%)	21 (44.68%)	21 (42%)	0.798
The number of HbA1c measurements	5.4 ± 0.14	5.4 ± 0.12	5.43 ± 0.13	0.90
Duration (y)	10 (5, 15)	10 (6, 20)	15 (9, 20)^a^^,^^b^	0.024
SBP (mmHg)	134.20 ± 14.31	133.67 ± 18.65	140.49 ± 24.02	0.759
DBP (mmHg)	78.76 ± 9.91	77.53 ± 11.60	77.04 ± 11.61	0.140
LDL (mmol/L)	1.78 (1.36, 2. 16)	1.97 (1.51, 2.61)	2.38 (1.78, 2.78)	0.402
EGFR (mL/min/1.73 m^2^)	95.73 ± 4.6	93.37 ± 5.91	88.26 ± 4.08	0.39
Triglyceride (mmol/L)	2.14 ± 0.32	2.02 ± 0.34	2.06 ± 0.14	0.942
Insulin (%)	21 (42%)	27 (57.44%)	38 (76%)	0.002
BMI (kg/m^2^)	24.6 (23, 26.57)	25.7 (23.59, 27.68)	24.61 (23.34, 26.12)	0.102
HbA1c	6.92 (6.42, 7.38)	7.62 (6.98, 8.36)^a^	7.94 (7.19, 8.91)^a^	< 0.001
HbA1cTIR	0.75 (0.53, 0.92)	0.43 (0.25, 0.66)^a^	0.09 (0, 0.3)^a^^,^^b^	< 0.001
SD-HbA1c	0.44 (0.32, 0.62)	0.62 (0.38, 0.88)^a^	0.69 (0.42, 1.19)^a^	0.001
CV-HbA1c	0.07 (0.05, 0.09)	0.08 (0.05, 0.10)	0.085 (0.06, 0.14)^a^	0.026
VIM-HbA1c	0.61 (0.47, 0.86)	0.65 (0.45, 0.99)	0.74 (0.59, 1.16)	0.115

*Note:* The centiles of the Gensini score were divided into three groups according to the centile method. Group T1: Gensini score < 20; group T2: Gensini score 20–50; in group T3, the continuous variables with the Gensini score > 50 were expressed as the mean standard deviation and compared by analysis of variance. If the data did not conform to the normal distribution, it was expressed as the median (interquartile range) and compared with the nonparametric test. Categorical variables were expressed as frequency (percentage) and compared using a chi-square test. Values are expressed as *n* (%) or median (interquartile range).

Abbreviations: BMI, body mass index; CV, coefficient of variation; DBP, diastolic blood pressure; EGFR, estimated glomerular filtration; Hb1Ac, glycosylated hemoglobin; HbA1cTIR, HbA1c time in range; LDL, low-density lipoprotein cholesterol; SBP, systolic blood pressure; SD, standard deviation; VIM, variation independent of mean.

^a^Statistically significant difference compared to the T1 group.

^b^Statistically significant difference compared to the T2 group.

**Table 2:** Relationship between glycosylated hemoglobin variability and the number of coronary artery lesion branches.

	**Model 1**		**Model 2**		**Model 3**	
	**OR**	**r**	**OR**	**r**	**OR**	**r**
HbA1cTIR	0.09 (0.03, 0.24)	< 0.001	0.11 (0.04, 0.31)	< 0.001	0.13 (0.04, 0.41)	< 0.001
SD-HbA1c	2.59 (1.32, 5. 11)	0.007	2.84 (1.34, 6.05)	0.002	2.38 (0.98, 5.77)	0.058
CV-HbA1c	1.26 (0.96, 1.64)	0.093	1.32 (0.99, 1.76)	0.017	1.32 (0.991, 1.76)	0.120
VIM-HbA1c	1.77 (0.84, 3.70)	0.142	2.39 (1.08, 5.29)	0.019	2.60 (1.15, 5.90)	0.026

*Note:* The correlation between the glycosylated hemoglobin variability and the number of coronary artery lesion branches was studied by multivariate logistic regression analysis. In Model 1, no factors were adjusted. In Model 2, sex, age, duration, SBP, DBP, LDL, and use of insulin using were adjusted. Model 3 is adjusted for factors in Model 2 and HbA1c.

Abbreviations: CV, coefficient of variation; Hb1Ac, glycosylated hemoglobin; HbA1cTIR, HbA1c time in range; OR, odds ratio; SD, standard deviation; VIM, variation independent of mean.

**Table 3:** Linear regression analyses for the Gensini score.

	**Unadjusted**		**Adjusted**	
	**β**	**r**	**β**	**r**
HbA1cTIR	−0.629	< 0.001	−0.576	< 0.001
SD-HbA1c	0.271	0.001	0.217	0.024
CV-HbA1c	0.176	0.033	0.160	0.052
VIM-HBA1c	0.129	0.120	0.181	0.022

*Note:* The adjusted linear logistic regression was adjusted for age, sex, disease duration, systolic blood pressure, diastolic blood pressure, low-density lipoprotein cholesterol, insulin use, and HbA1c.

Abbreviations: CV, coefficient of variation; HbA1c, glycosylated hemoglobin; HbA1cTIR, HbA1c time in range; SD, standard deviation; VIM, variation independent of mean.

## Data Availability

The patient information data used to support the findings of this study are restricted by the Medical Ethics Committee of Shengjing Hospital affiliated to China Medical University in order to protect patient privacy. Data are available from Na Wu, 3441535223@qq.com, for researchers who meet the criteria for access to confidential data.

## Supporting Information

Supporting Information Additional supporting information can be found online in the Supporting Information section. Table S1 HbA1c targets different populations. Table S2 Coronary artery stenosis according to tertile of HbA1c variability. Table S3 Multivariate analysis for the Gensini score. Table S4a HbA1c ≤ 7% multivariate analysis for the number of involved vessels.Table S4b HbA1c ≤ 7% linear regression analyses for the Gensini score. Table S4c HbA1c > 7% multivariate analysis for the number of involved vessels. Table S4d HbA1c > 7% linear regression analyses for the Gensini score.

## References

[1] Chinese Diabetes Society (2021). Guidelines for the prevention and control of type 2 diabetes in China (2020 Edition). *Chinese Journal of Practical Internal Medicine*.

[2] Zimmet P. Z., Magliano D. J., Herman W. H., Shaw J. E. (2014). Diabetes: a 21st century challenge. *The Lancet Diabetes and Endocrinology*.

[3] Hayward R. A., Reaven P. D., Wiitala W. L. (2015). Follow-up of glycemic control and cardiovascular outcomes in type 2 diabetes. *New England Journal of Medicine*.

[4] Ghosh-Swaby O. R., Goodman S. G., Leiter L. A. (2020). Glucose-lowering drugs or strategies, atherosclerotic cardiovascular events, and heart failure in people with or at risk of type 2 diabetes: an updated systematic review and meta-analysis of randomised cardiovascular outcome trials. *The Lancet Diabetes and Endocrinology*.

[5] American Diabetes Association (2019). 2. Classification and diagnosis of Diabetes:Standards of medical care in diabetes-2019. *Diabetes Care*.

[6] Davies M. J., D'Alessio D. A., Fradkin J. (2018). Management of hyperglycemia in type 2 diabetes, 2018. A consensus report by the American Diabetes Association (ADA) and the European Association for the Study of Diabetes (EASD). *Diabetes Care*.

[7] Zoungas S., Chalmers J., Neal B. (2014). Follow-up of blood-pressure lowering and glucose control in type 2 diabetes. *The New England Journal of Medicine*.

[8] Duckworth W., Abraira C., Moritz T. (2009). Glucose control and vascular complications in veterans with type 2 diabetes. *New England Journal of Medicine*.

[9] Diabetes Control and Complications Trial (DCCT)/Epidemiology of Diabetes Interventions and Complications (EDIC) Study Research Group (2016). Intensive diabetes treatment and cardiovascular outcomes in type 1 diabetes: the DCCT/EDIC study 30-year follow-up. *Diabetes Care*.

[10] Xia J., Xu J., Li B. (2017). Association between glycemic variability and major adverse cardiovascular and cerebrovascular events (MACCE) in patients with acute coronary syndrome during 30-day follow-up. *Clinica Chimica Acta*.

[11] Matsutani D., Sakamoto M., Iuchi H. (2018). Glycemic variability in continuous glucose monitoring is inversely associated with baroreflex sensitivity in type 2 diabetes: a preliminary report. *Cardiovascular Diabetology*.

[12] Takahashi H., Iwahashi N., Kirigaya J. (2018). Glycemic variability determined with a continuous glucose monitoring system can predict prognosis after acute coronary syndrome. *Cardiovascular Diabetology*.

[13] Hirakawa Y., Arima H., Zoungas S. (2014). Impact of visit-to-visit glycemic variability on the risks of macrovascular and microvascular events and all-cause mortality in type 2 diabetes: the ADVANCE trial. *Diabetes Care*.

[14] Li S., Nemeth I., Donnelly L., Hapca S., Zhou K., Pearson E. R. (2020). Visit-to-visit HbA_1c_ variability is associated with cardiovascular disease and microvascular complications in patients with newly diagnosed type 2 diabetes. *Diabetes Care*.

[15] Cardoso C. R. L., Leite N. C., Moram C. B. M., Salles G. F. (2018). Long-term visit-to-visit glycemic variability as predictor of micro- and macrovascular complications in patients with type 2 diabetes: the Rio de Janeiro type 2 diabetes cohort study. *Cardiovascular Diabetology*.

[16] Sheng C. S., Tian J., Miao Y. (2020). Prognostic significance of long-term HbA_1c_ variability for all-cause mortality in the ACCORD trial. *Diabetes Care*.

[17] Yang C. Y., Su P. F., Hung J. Y., Ou H. T., Kuo S. (2020). Comparative predictive ability of visit-to-visit HbA_1c_ variability measures for microvascular disease risk in type 2 diabetes. *Cardiovascular Diabetology*.

[18] Ghouse J., Skov M. W., Kanters J. K. (2019). Visit-to-visit variability of hemoglobin A1c in people without diabetes and risk of major adverse cardiovascular events and all-cause mortality. *Diabetes Care*.

[19] Battelino T., Danne T., Bergenstal R. M. (2019). Clinical targets for continuous glucose monitoring data interpretation: recommendations from the international consensus on time in range. *Diabetes Care*.

[20] Prentice J. C., Mohr D. C., Zhang L. (2021). Increased hemoglobin A1c time in range reduces adverse health outcomes in older adults with diabetes. *Diabetes Care*.

[21] Ravipati G., Aronow W. S., Ahn C., Sujata K., Saulle L. N., Weiss M. B. (2006). Association of hemoglobin A1c level with the severity of coronary artery disease in patients with diabetes mellitus. *The American Journal of Cardiology*.

[22] Selvin E., Wattanakit K., Steffes M. W., Coresh J., Sharrett A. R. (2006). HbA_1c_ and peripheral arterial disease in Diabetes. *Diabetes Care*.

[23] Engoren M., Habib R. H., Zacharias A. (2008). The prevalence of elevated hemoglobin A1c in patients undergoing coronary artery bypass surgery. *Journal of Cardiothoracic Surgery*.

[24] Wang X., Han Z., Hao G., Li Y., Dong X., Wang C. (2015). Hemoglobin A1c level is not related to the severity of atherosclerosis in patients with acute coronary syndrome. *Disease Markers*.

[25] Zhu D. (2020). Expert consensus on glycated hemoglobin A1C targets and management algorithm for Chinese adults with type 2 diabetes. *China Medical Abstracts*.

[26] Gensini G. G. (1983). A more meaningful scoring system for determining the severity of coronary heart disease. *The American Journal of Cardiology*.

[27] Guo K., Zhao Q., Wang M. (2022). The scope of HbA_1c_ variability and risk of vascular complications among patients with type 2 diabetes: a systematic review and meta-analysis of prospective studies. *Hormone and Metabolic Research*.

[28] Yang C. D., Shen Y., Lu L. (2020). Visit-to-visit HbA_1c_ variability is associated with in-stent restenosis in patients with type 2 diabetes after percutaneous coronary intervention. *Cardiovascular Diabetology*.

[29] Chung H. S., Hwang S. Y., Kim J. A. (2022). Implications of fasting plasma glucose variability on the risk of incident peripheral artery disease in a population without diabetes: a nationwide population-based cohort study. *Cardiovascular Diabetology*.

[30] Ceriello A., Lucisano G., Prattichizzo F. (2022). HbA_1c_ variability predicts cardiovascular complications in type 2 diabetes regardless of being at glycemic target. *Cardiovascular Diabetology*.

[31] Noyes J. D., Soto-Pedre E., Donnelly L. A., Pearson E. R. (2018). Characteristics of people with high visit-to-visit glycaemic variability in type 2 diabetes. *Diabetic Medicine*.

[32] Pieber T. R., Marso S. P., McGuire D. K. (2018). DEVOTE 3: temporal relationships between severe hypoglycaemia, cardiovascular outcomes and mortality. *Diabetologia*.

[33] Farabi S. S., Carley D. W., Smith D., Quinn L. (2015). Impact of exercise on diurnal and nocturnal markers of glycaemic variability and oxidative stress in obese individuals with type 2 diabetes or impaired glucose tolerance. *Diabetes & Vascular Disease Research*.

[34] Monnier L., Colette C., Mas E. (2010). Regulation of oxidative stress by glycaemic control: evidence for an independent inhibitory effect of insulin therapy. *Diabetologia*.

[35] Chang C. M., Hsieh C. J., Huang J. C., Huang I. C. (2012). Acute and chronic fluctuations in blood glucose levels can increase oxidative stress in type 2 diabetes mellitus. *Acta Diabetologica*.

[36] Tateishi K., Saito Y., Kitahara H., Kobayashi Y. (2022). Impact of glycemic variability on coronary and peripheral endothelial dysfunction in patients with coronary artery disease. *Journal of Cardiology*.

[37] Cosentino-Gomes D., Rocco-Machado N., Meyer-Fernandes J. R. (2012). Cell signaling through protein kinase C oxidation and activation. *International Journal of Molecular Sciences*.

[38] Gude F., Díaz-Vidal P., Rúa-Pérez C. (2017). Glycemic variability and its association with demographics and lifestyles in a general adult population. *Journal of Diabetes Science and Technology*.

[39] Ratter J. M., Rooijackers H. M., Tack C. J. (2017). Proinflammatory effects of hypoglycemia in humans with or without diabetes. *Diabetes*.

[40] Gogitidze Joy N., Hedrington M. S., Briscoe V. J., Tate D. B., Ertl A. C., Davis S. N. (2010). Effects of acute hypoglycemia on inflammatory and pro-atherothrombotic biomarkers in individuals with type 1 diabetes and healthy individuals. *Diabetes Care*.

[41] Reno C. M., Daphna-Iken D., Chen Y. S., VanderWeele J., Jethi K., Fisher S. J. (2013). Severe hypoglycemia-induced lethal cardiac arrhythmias are mediated by sympathoadrenal activation. *Diabetes*.

