# Supplementary material for: Relationship Between Glycosylated Hemoglobin Variability and the Severity of Coronary Artery Disease in Patients With Type 2 Diabetes Mellitus
Source: J Diabetes Res. 2024 Aug 1;2024:9958586. doi: 10.1155/2024/9958586 (PMC11309811; doi:10.1155/2024/9958586)
Supplement: Supporting Information — Additional supporting information can be found online in the Supporting Information section. Table S1 HbA1c targets different populations. Table S2 Coronary artery stenosis according to tertile of HbA1c variability. Table S3 Multivariate analysis for the Gensini score. Table S4a HbA1c ≤ 7% multivariate analysis for the number of involved vessels.Table S4b HbA1c ≤ 7% linear regression analyses for the Gensini score. Table S4c HbA1c > 7% multivariate analysis for the number of involved vessels. Table S4d HbA1c > 7% linear regression analyses for the Gensini score. [file 9958586.f1.doc]

**Supplementary Table S1** **HbA1c targets different populations.**

| **Target** | **Population** |
| --- | --- |
| ≤6.5% | Young patients, short diabetes duration, long life expectancy, and no complications. |
| ≤7% | Most adult T2DM patients. |
| 7– 8% | Patients with long diabetes duration, with CVD or extremely high-risk CVD; older patients without drugs that increase the risk of hypoglycemia with moderate impairment of health (combined with multiple chronic diseases, impairment of more than two daily activities or mild to moderate cognitive  impairment) |
| 7.5–8% | Older patients using drugs that increase the risk of hypoglycemia (such as insulin) with moderate impairment of health (combined with multiple chronic diseases, impairment of more than two daily activities, or mild to moderate cognitive impairment). |
| 6.5–7.5% | Most patients with T2DM complicated with CKD under the age of 40, patients with CKD1-2 over the age of 40, and relatively healthy older patients (less complicated chronic diseases, good physical  function, and complete cognitive function) without drugs that increase the risk of hypoglycemia. |
| 7–7.5% | CKD3-4 patients without insulin, relatively healthy older patients (less complicated chronic diseases, good physical function, and complete cognitive function) with drugs that increase the risk of  Hypoglycemia. |
| 7.5–8.5% | CKD3-4 patients using insulin and CKD5 patients receiving dialysis. |
| 8–9% | Older patients with poor health status (such as end-stage chronic disease, long-term need for care from others), patients with malignant tumors, Alzheimer’s disease, epilepsy, and life expectancy of less than 5 years, or patients with mental or intellectual disorders, which lead to  difficulties in implementing treatment plans. |

CKD, chronic kidney disease; CVD, cardiovascular disease; Hb1Ac, glycosylated hemoglobin; T2DM, type 2 diabetes mellitus.

**Supplementary** **Table S2** **Coronary artery stenosis according to tertile of HbA1c variability.**

| **HbA1cTIR** | | | | |
| --- | --- | --- | --- | --- |
|  | T1 (n=50) | T2 (n=49) | T3 (n=48) | *r* |
| Multivessel disease | 43 (86%) | 37 (75.51%) | 25 (52.08%)***#** | 0.004 |
| 3-vessel disease | 26 (52%) | 14 (28.57%)* | 9 (18.75%)* | 0.001 |
| Gensini score | 64 (43, 88) | 28 (16, 44)* | 10 (6, 28)***#** | <0.001 |
| **VIM-HbA1c** | | | | |
|  | T1 (n=48) | T2 (n=50) | T3 (n=49) | *r* |
| Multivessel disease | 30 (62.5%) | 41 (82%) | 34 (69.39%) | 0.096 |
| 3-vessel disease | 13 (27.08%) | 17 (34%) | 19 (38.78%) | 0.473 |
| Gensini score | 28 (8, 44) | 40 (14, 58) | 40 (16, 62) | 0.185 |
| **SD-HbA1c** | | | | |
|  | T1 (n=50) | T2 (n=50) | T3 (n=47) | *r* |
| Multivessel disease | 30 (60%) | 38 (76%) | 37 (78.72%) | 0.086 |
| 3-vessel disease | 12 (24%) | 17 (34%) | 20 (42.55%) | 0.154 |
| Gensini score | 24 (6, 50) | 28 (14, 52) | 48 (28, 74)*# | 0.008 |
| **CV-HbA1c** | | | | |
|  | T1 (n=48) | T2 (n=50) | T3 (n=49) | *r* |
| Multivessel disease | 31 (64.58%) | 38 (76%) | 36 (73.47%) | 0.427 |
| 3-vessel disease | 13 (27.08%) | 17 (34%) | 19 (38.78%) | 0.473 |
| Gensini score | 24 (8, 54) | 28 (14, 50) | 42 (24, 64)*# | 0.036 |

Note: The interquartile of HbA1c variability was divided into 3 groups according to the interquartile method

Group T1: ＜P33; Group T2: P33-P66; Group T3: p >66

CV, coefficient of variation; Hb1Ac, glycosylated hemoglobin; HbA1cTIR, HbA1c time in range; SD, standard

deviation; VIM, Variation independent of mean value.

*Represents a statistically significant difference compared to the T1 group

#Represents a statistically significant difference compared to the T2 group

**Supplementary Table S3 Multivariate analysis for Gensini score.**

|  | Model 1 | Model 2 | | Model 3 | |  |
| --- | --- | --- | --- | --- | --- | --- |
|  | OR | *r* | OR | *r* | OR | *r* |
| HbA1cTIR | 0.007 (0.002, 0.03) | <0.001 | 0.008 (0.002, 0.03) | <0.001 | 0.01 (0.002, 0.04) | ＜0.001 |
| SD-HbA1c | 4. 15 (2.03, 8.48) | <0.001 | 4.45 (2.03, 9.78) | <0.001 | 4. 12 (1.64, 10.35) | 0.001 |
| CV-HbA1c | 1.42 (1.08, 1.86) | 0.004 | 1.49 (1.12, 1.99) | <0.001 | 1.41 (1.04, 1.92) | 0.007 |
| VIM-HbA1c | 2.10 (1.01, 4.36) | 0.036 | 2.65 (1.20, 5.84) | 0.005 | 3.26 (1.43, 7.47) | 0.003 |

Note: the correlation between the variability of HbA1c and the three-position of Gensini score was studied by multivariate logistic regression analysis.

HbA1c: glycated hemoglobin; SD: standard deviation; CV: coefficient of variation; VIM: variation independent of mean

In Model 1, no factors were adjusted. In Model 2, sex, age, duration, SBP, DBP, LDL, and use of insulin using were adjusted. In Model 3, adjusted for factors in Model 2 and HbA1c.

**Supplementary Table S4a** **HbA1c ≤7% multivariate analysis for the number of involved**

**vessels.**

|  | **Model 1** | | **Model 2** | | **Model 3** | |
| --- | --- | --- | --- | --- | --- | --- |
|  | OR | r | OR | r | OR | r |
| HbA1c TIR | 0.007 (0.002, 0.03) | <0.001 | 0.01 (0.001, 0.10) | <0.001 | 0.008 (0.001, 0.08) | <0.001 |
| SD-HbA1c | 4.15 (2.03, 8.48) | 0.63 | 6.13 (0.50, 75.55) | 0.18 | 7.01 (0.52, 87.71) | 0.17 |
| CV- HbA1c | 1.42 (1.08, 1.86) | 0.20 | 1.26 (0.77, 2.05) | 0.89 | 1.26 (0.77, 2.06) | 0.88 |
| VIM- HbA1c | 2.10 (1.01, 4.36) | 0.31 | 3.60 (0.83, 15.60) | 0.10 | 3.72 (0.84, 16.57) | 0.10 |

CV, coefficient of variation; Hb1Ac, glycosylated hemoglobin; HbA1cTIR, HbA1c time in range; OR, odds ratio;

SD, standard deviation; VIM, Variation independent of mean value.

In Model 1, no factors were djusted. In Model 2, sex, age, duration of illness, and low-density lipoprotein

cholesterol were adjusted.

Model 3 was adjusted for the factors in Model 2 and HbA1c.

**Supplementary Table S4b HbA1c ≤7% linear regression analyses for the Gensini score.**

|  | **Unadjusted** | **P** | **Adjusted** |  |
| --- | --- | --- | --- | --- |
|  | **β** |  | **β** | **P** |
| HbA1cTIR | −0.713 | <0.001 | −0.749 | <0.001 |
| SD-HbA1c | 0.020 | 0.890 | 0.128 | 0.412 |
| CV- HbA1c | 0.055 | 0.697 | 0.010 | 0.945 |
| VIM-HbA1c | 0.091 | 0.523 | 0.145 | 0.338 |

CV, coefficient of variation; HbA1c, glycosylated hemoglobin; HbA1cTIR, HbA1c time in range; SD, standard

deviation; VIM, Variation independent of mean value.

The adjusted linear logistic regression adjusted for age, sex, duration of illness, low-density lipoprotein cholesterol, and HbA1c.

**Supplementary Table S4c HbA1c >7% multivariate analysis for the number of involved vessels.**

|  | **Model 1** | | **Model 2** | | **Model 3** | |
| --- | --- | --- | --- | --- | --- | --- |
|  | OR | r | OR | r | OR | r |
| HbA1c TIR | 0.20 (0.06, 0.70) | 0.009 | 0.26 (0.07, 0.97) | 0.035 | 0.40 (0.09, 1.78) | 0.218 |
| SD-HbA1c | 1.72 (0.79, 3.77) | 0.175 | 2.17 (0.91, 5.13) | 0.079 | 1.67 (0.65, 4.28) | 0.386 |
| CV- HbA1c | 1.12 (0.80, 1.59) | 0.275 | 1.22 (0.85, 1.75) | 0.133 | 1.16 (0.80, 1.68) | 0.255 |
| VIM- HbA1c | 1.31 (0.53, 3.24) | 0.562 | 1.67 (0.62, 4.52) | 0.315 | 1.82 (0.67, 4.99) | 0.247 |

CV, coefficient of variation; HbA1c, glycosylated hemoglobin; HbA1cTIR, HbA1c time in range; OR, odds ratio; SD, standard deviation; VIM, variation independent of mean value.

In Model 1, no factors were adjusted, Model 2 adjusted for sex, age, duration, and low-density lipoprotein cholesterol level. Model 3 was adjusted for the factors in Model 2 and HbA1c.

**Supplementary Table S4d HbA1c >7% linear regression analyses for the Gensini score.**

|  | **Unadjusted** | *r* | **Adjusted** |  |
| --- | --- | --- | --- | --- |
|  | β |  | β | *r* |
| HbA1cTIR | -0.576 | <0.001 | -0.595 | <0.001 |
| SD-HbA1c | 0.215 | 0.037 | 0.175 | 0.116 |
| CV- HbA1c | 0.179 | 0.082 | 0.167 | 0.106 |
| VIM-HbA1c | 0.105 | 0.312 | 0.155 | 0.126 |

CV, coefficient of variation; HbA1c, glycosylated hemoglobin; HbA1cTIR, HbA1c time in range; SD, standard deviation; VIM, Variation independent of mean value.

The adjusted linear logistic regression adjusted for age, sex, duration, low-density lipoprotein cholesterol, and HbA1c
